# Acid ceramidase ASAH1 is a key regulator of epidermal ceramide levels and composition

**DOI:** 10.1016/j.jbc.2026.111178

**Published:** 2026-01-20

**Authors:** Wakana Nobumoto, Tatsuro Naganuma, Nana Nozaka, Yusuke Ohno, Koki Nojiri, Akio Kihara

**Affiliations:** Laboratory of Biochemistry, Faculty of Pharmaceutical Sciences, Hokkaido University, Sapporo, Japan

**Keywords:** ceramidase, ceramide, enzyme kinetics, epidermis, gene KO, lipid, liposome, mass spectrometry, metabolism, sphingolipid

## Abstract

Maintenance of appropriate ceramide levels and composition in the stratum corneum of the epidermis is essential for skin barrier function. Although ceramide homeostasis is regulated by both synthesis and degradation, the extent of ceramide degradation in the epidermis, as well as the ceramidase responsible for this degradation, has thus far remained unclear. Here, we found that the acid ceramidase ASAH1 is strongly expressed in differentiated human keratinocytes. To investigate its role, we generated *ASAH1* KO cells using immortalized human keratinocytes and analyzed their ceramide levels. Under differentiation conditions, *ASAH1* KO keratinocytes exhibited a marked accumulation of ceramide classes composed of sphingosine (S) or dihydrosphingosine (dS) and nonhydroxy fatty acid (N) (ceramides NS and NdS). In contrast, ceramides with (an) additional hydroxyl group(s)—such as those containing phytosphingosine (P) or 6-hydroxysphingosine (H) and N or α-hydroxy fatty acid (A) (ceramides NP, NH, AP, and AH)—showed a moderate or no increase. Similar results were obtained upon treatment with SABRAC, a specific ASAH1 inhibitor. *In vitro* enzyme assays revealed that ASAH1 exhibited strong activity toward NS and NdS, weak activity toward NP and NH, and no activity toward AP. These results indicate that ASAH1-mediated ceramide class-dependent degradation occurs in differentiated human keratinocytes. This degradation likely plays an important role in maintaining appropriate ceramide levels and class composition in the stratum corneum, thereby contributing to the integrity of the skin barrier.

The epidermis serves as a permeability barrier (skin barrier) that prevents transepidermal water loss and protects the body from external substances, including pathogens, allergens, and chemicals. Reduced skin barrier function increases the risk of or causes several cutaneous disorders, such as infections, atopic dermatitis, xerosis, and ichthyosis (epidermal differentiation disorders) (1, 2, 3). The epidermis is composed of the following four layers, in order from innermost to outermost: stratum basale, stratum spinosum, stratum granulosum, and stratum corneum. Of these, the stratum corneum plays the most important role in skin barrier formation. In this layer, intercellular spaces between terminally differentiated keratinocytes (corneocytes) are filled with multilayered lipid structures known as lipid lamellae (4, 5). The major components of the lipid lamellae are ceramides, cholesterol, and free fatty acids (FAs) (6, 7, 8).

Keratinocytes, which constitute the majority of cells in the epidermis, proliferate in the stratum basale and migrate outward while differentiating into cells with morphology and functions specific to each epidermal layer. Most of the ceramides in the stratum corneum are derived from the stratum granulosum, where they are produced in the endoplasmic reticulum, converted to complex sphingolipids (mainly glucosylceramides and partly sphingomyelins) in the Golgi apparatus, transported to and temporarily stored in lamellar bodies, and secreted into the extracellular space at the boundary between the stratum granulosum and the stratum corneum (5, 9, 10). Around the time of this secretion, the polar head groups of the complex sphingolipids are removed, converting them back into ceramides (9).

Ceramides consist of a long-chain base and an FA (*N*-acyl chain). Human ceramides are classified into 25 classes, each consisting of a different combination of five long-chain bases (sphingosine [S], dihydrosphingosine [dS], phytosphingosine [P], 6-hydroxysphingosine [H], and 4,14-sphingadiene [Sd]) and five FAs (nonhydroxy FA [N], α-hydroxy FA [A], ω-hydroxy FA [O], esterified ω-hydroxy FA [EO], and protein-bound FA [PB-]) (5, 9, 11, 12) (Fig. 1, *A* and *B*). Each ceramide class is represented by the combination of the abbreviations for their FA and long-chain base (*e.g.*, NS, EOS) (Fig. 1*C*). In the stratum corneum, most of these ceramide classes are components of the lipid lamellae, but exceptionally, PB-containing ceramides (protein-bound ceramides) are components of the corneocyte lipid envelope, a plasma membrane–like structure of corneocytes (12, 13, 14). Protein-bound ceramides are covalently bound to corneocyte surface proteins (cornified envelope proteins) (12, 15). Ceramides other than protein-bound ceramides (free ceramides; non–protein-bound ceramides) are divided into conventional-type ceramides and ω-*O*-acylceramides (hereafter referred to simply as acylceramides), which contain EO (EOS, EOdS, EOP, EOH, and EOSd). In acylceramides, an *O*-acyl chain (mainly linoleic acid) is attached to the ω-terminal hydroxyl group of the *N*-acyl chain *via* an ester bond (5, 9, 16). Each ceramide class contains multiple species with different carbon-chain lengths in the long-chain base and FA moieties (as well as different degrees of unsaturation in the FA moiety). We have reported that 23 classes and 1581 species of ceramides exist in the human stratum corneum (11). While the majority of ceramides in most tissues are of the NS type, the human stratum corneum predominantly contains ceramide species with additional hydroxyl groups, such as NP and NH (each possessing one more hydroxyl group than NS) and AP and AH (each possessing two more). These species account for 29% (NP), 23% (NH), 9% (AH), and 6% (AP) of total free ceramides, respectively (11). The maintenance of ceramide class composition is critical for the formation of an intact skin barrier. For example, in atopic dermatitis, the proportions of NP, NH, EOS, EOH, and EOP are reduced, whereas those of NS and AS are increased (17, 18, 19). Impaired production of acylceramides and/or protein-bound ceramides leads to congenital ichthyosis (2).

**Figure 1:**
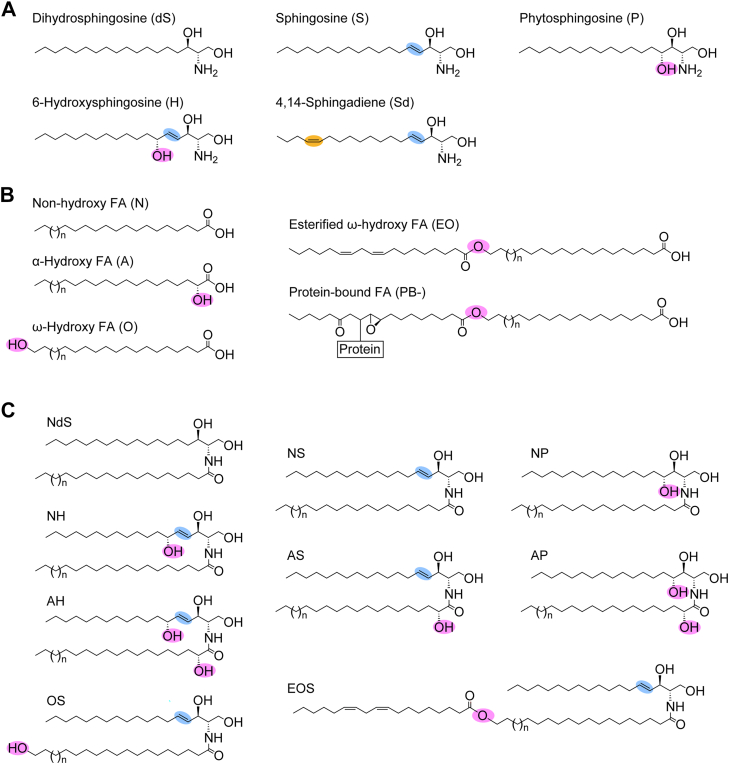
**Structure of ceramides.***A* and *B*, structures of the long-chain base (*A*) and FA (*B*) components that constitute human ceramides. *C*, structures of ceramide classes analyzed in this study. The characteristic functional groups of each long-chain base or FA (*N*-acyl chain) are highlighted in color. FA, fatty acid.

Ceramidases catalyze the cleavage of the amide bond in ceramide, converting it into a long-chain base and an FA. Ceramidases are classified into acid, neutral, and alkaline ceramidases depending on the optimum pH for enzymatic activity. Mammals have acid ceramidase ASAH1, neutral ceramidase ASAH2, and alkaline ceramidases ACER1, ACER2, and ACER3 (20, 21). ASAH1 is localized in lysosomes and is present in a wide range of tissues (22). Mutations in *ASAH1* cause Farber's disease, which is characterized by subcutaneous nodules, arthritis-like symptoms, and hoarseness (21, 23, 24, 25). *Asah1* KO mice exhibit embryonic lethality during early developmental stages (26, 27). ASAH2 is primarily localized in the plasma membrane and is expressed in many tissues, including the small intestine (28, 29, 30). Because ceramides are poorly absorbed by tissues and cells, dietary ceramides are hydrolyzed by ASAH2 into long-chain bases and FAs prior to absorption in the small intestine (30). ACER1–3 are localized in the endoplasmic reticulum and/or the Golgi apparatus, with ACER1 expressed in the skin, ACER2 in the placenta, pancreas, and heart, and ACER3 broadly expressed across various tissues, with particularly high expression in the placenta (20, 21, 31, 32, 33, 34, 35). *Acer1* KO mice exhibit a slight increase in ceramide levels in the skin (approximately 1.2-fold those in wild type mice), abnormalities in hair shaft cuticle formation, and cyclic alopecia (35).

So far, the extent of ceramide degradation in the epidermis, its physiological significance, and the ceramidase isozyme(s) involved have remained unclear. In this study, we revealed that the acid ceramidase ASAH1 actively degrades specific classes of ceramides in differentiated keratinocytes. ASAH1 is highly active toward ceramides with fewer hydroxyl groups (NS and NdS) but shows low or no activity toward ceramides with more hydroxyl groups (NP, NH, and AP). The class-dependent degradation of ceramides by ASAH1 likely plays a key role in preserving the optimal ceramide composition necessary for constructing lipid lamellae with strong barrier properties.

## Results

### Strong expression of *ACER1* and *ASAH1* in differentiated keratinocytes

**Figure 2:**
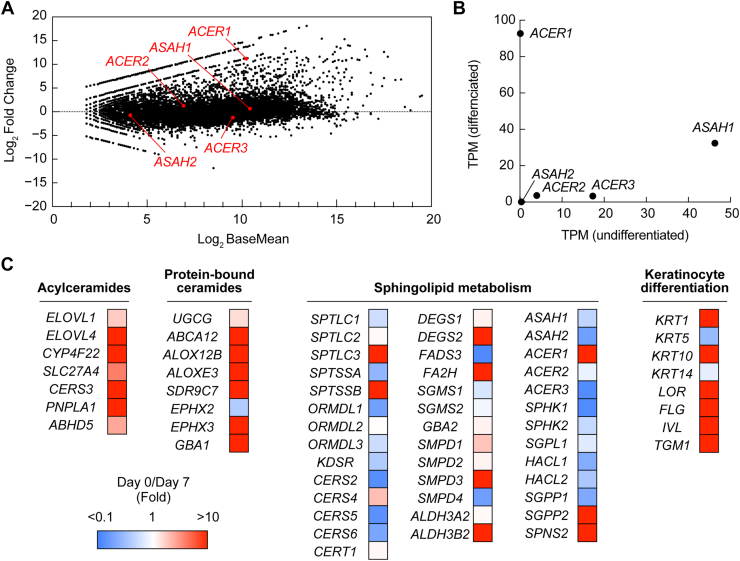
**Differentiation-dependent expression of ceramidases in human primary keratinocytes.** Total RNA was prepared from undifferentiated and 7-day differentiated primary human keratinocytes, followed by RNA sequencing analysis. *A*, mean average (MA) plot showing gene expression changes between undifferentiated and differentiated keratinocytes. In this plot, the log_2_ fold changes (*y*-axis) are plotted against the log_2_ base mean expression levels (*x*-axis). Ceramidase genes are indicated by *red dots*. *B*, TPM values of individual ceramidase genes in undifferentiated (*x*-axis) and 7-day differentiated (*y*-axis) keratinocytes. *C*, heatmap illustrating the differences in expression of genes involved in the synthesis of acylceramides and protein-bound ceramides, genes related to sphingolipid metabolism, and keratinocyte differentiation markers. The color scale represents the ratio of TPM values in differentiated keratinocytes relative to those in undifferentiated keratinocytes. TPM, transcripts per million.

To investigate the expression levels of individual ceramidase genes before and after keratinocyte differentiation, we subjected undifferentiated and differentiated primary human keratinocytes to RNA sequencing analysis. In this analysis, approximately 15,000 genes were successfully mapped, and gene expression levels before and after differentiation were compared (Fig. 2*A* and Table S1). Of the ceramidases, *ASAH1* expression levels were high in both undifferentiated and differentiated keratinocytes (Fig. 2*B*). *ACER1* expression was barely detectable in undifferentiated keratinocytes but was strongly upregulated upon differentiation, exhibiting the highest expression levels among ceramidase genes in differentiated keratinocytes. *ACER3* showed moderate expression levels in undifferentiated keratinocytes, but its expression decreased upon differentiation. The expression levels of other ceramidase genes remained low in both undifferentiated and differentiated keratinocytes. Many genes involved in sphingolipid metabolism, particularly those responsible for the production of acylceramides and protein-bound ceramides, showed increased expression during keratinocyte differentiation (Fig. 2*C* and Table S2). Differentiation markers known to be expressed in the stratum spinosum and/or stratum granulosum also showed elevated expression levels in differentiated keratinocytes.

### Involvement of *ASAH1* in ceramide degradation in differentiated keratinocytes

**Figure 3:**
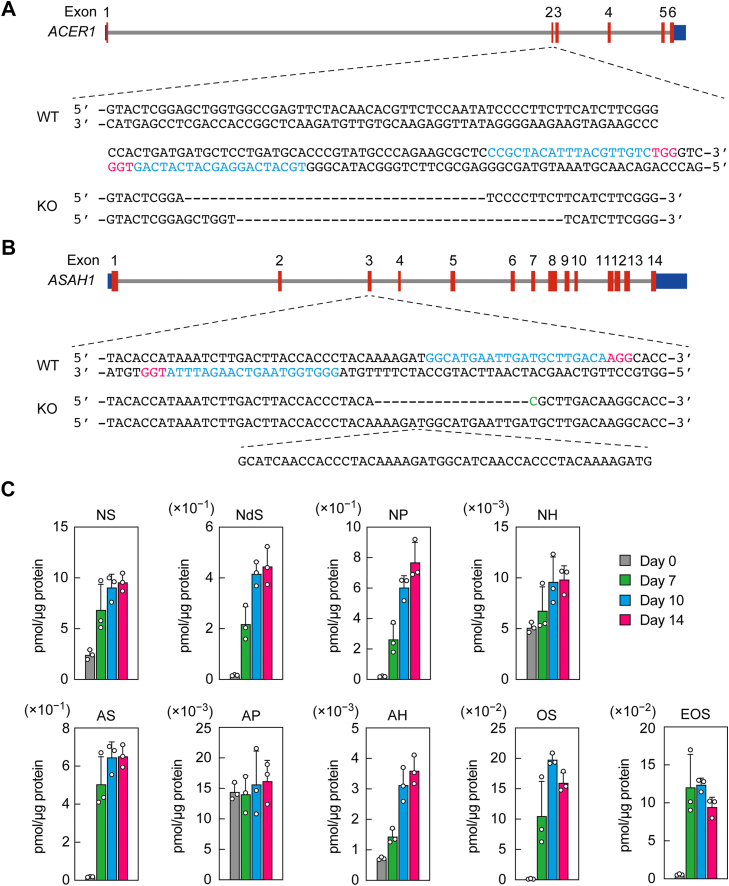
**Generation of *ACER1* and *ASAH1* KO keratinocytes and differentiation-dependent changes in ceramide composition.***A* and *B*, schematic representation of the human *ACER1* (*A*) and *ASAH1* (*B*) gene structures, along with the mutations introduced in the respective KO keratinocytes. CDSs are shown as *red boxes*, and untranslated regions are shown as *blue boxes*. Guide RNA target sequences are indicated in *blue*, protospacer-adjacent motif sequences in *red*, and missense mutations in *green*. Only the sense strands are shown for KO alleles. *C*, lipids were extracted from immortalized human keratinocytes (NHEK/SVTERT3-5) differentiated for 0, 7, 10, or 14 days, and the indicated ceramide classes were analyzed *via* LC–MS/MS. Values presented are mean + SD (n = 3). CDS, coding sequence.

To investigate the contribution of *ACER1* and *ASAH1*—both highly expressed in differentiated keratinocytes—to ceramide degradation, each gene was disrupted using the CRISPR–Cas9 system in immortalized human keratinocytes (NHEK/SVTERT3-5), which stably express telomerase reverse transcriptase and SV40 large T antigen. We obtained *ACER1* KO keratinocytes in which each allele contained a deletion of 35 bp or 38 bp within exon 2 (Fig. 3*A*). For *ASAH1*, KO keratinocytes were obtained in which each allele harbored distinct mutations within exon 3: one allele contained an 18-bp deletion and a single-base substitution, whereas the other carried a 48-bp insertion (Fig. 3*B*).

Before analyzing the ceramide composition in the KO keratinocytes generated, we first examined the ceramide profiles of the parental keratinocytes (NHEK/SVTERT3-5) using LC–MS/MS, tracking differentiation-induced alterations in ceramide composition over the course of the differentiation period. In this analysis, we measured the nine most abundant classes of free ceramides in the human stratum corneum: NS, NdS, NP, NH, AS, AP, AH, OS, and EOS (11) (Fig. 1*C*). Compared with levels at day 0 of differentiation, the quantities of NS, AS, and EOS ceramides had increased markedly by day 7 and remained relatively unchanged thereafter (Fig. 3*C*). The levels of NdS, NP, NH, AH, and OS ceramides had increased substantially by day 7 and further by day 10, with little additional change observed on day 14. In contrast, the levels of AP ceramides remained constant throughout the differentiation period.

**Figure 4:**
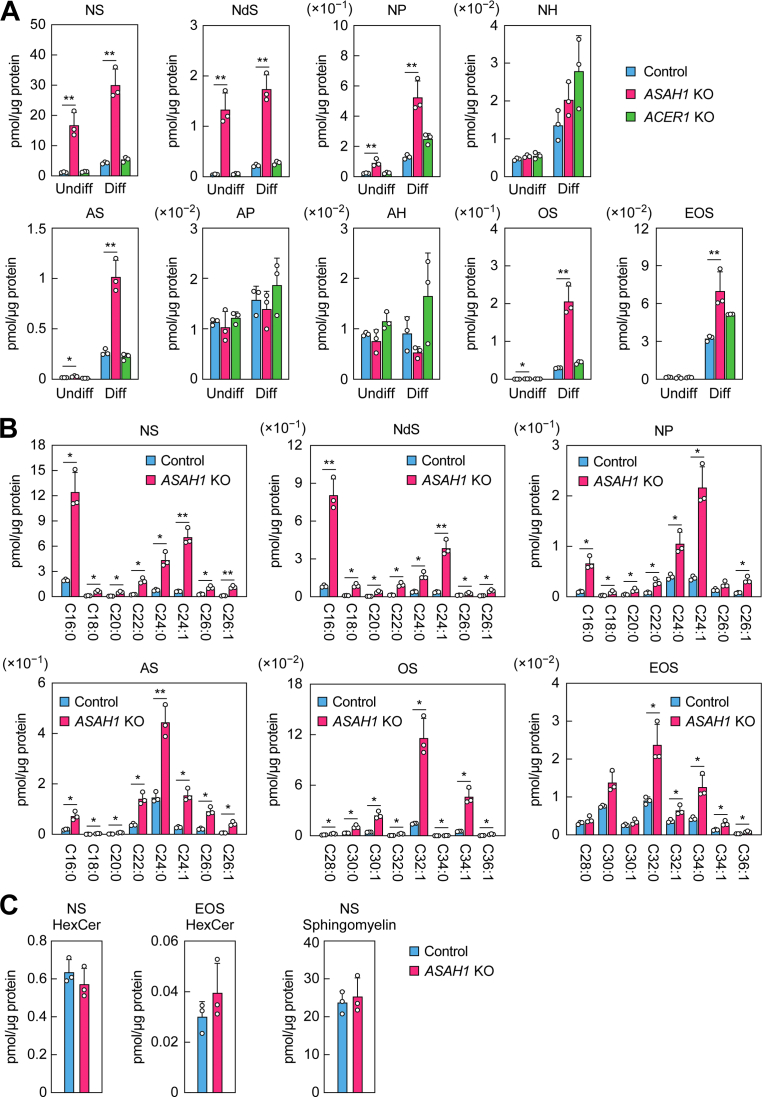
**Ceramide class–dependent accumulation in *ASAH1* KO keratinocytes.***A* and *B*, lipids were extracted from control (*A* and *B*), *ASAH1* KO (*A*–*C*), and *ACER1* KO (*A*) keratinocytes under undifferentiated conditions (*A*) and after 14 days of differentiation (*A*–*C*), and the indicated classes of ceramides (*A* and *B*), hexosylceramides (*C*), and sphingomyelins (*C*) were quantified *via* LC–MS/MS. Values presented are mean + SD of total quantities of the indicated lipid classes (*A* and *C*) and individual ceramide species categorized by FA moiety (*B*) (n = 3; ∗*p* < 0.05; ∗∗*p* < 0.01; Dunnett’s test *versus* control in *A*, Welch’s *t* test in *B*). Diff, differentiated; HexCer, hexosylceramide; Undiff, undifferentiated.

Next, we investigated the effects of *ACER1* or *ASAH1* KO on ceramide levels using undifferentiated and 14-day differentiated keratinocytes. In *ACER1* KO keratinocytes, the levels of all ceramide classes examined remained similar to those observed in control keratinocytes, regardless of differentiation status (Fig. 4*A*). These findings suggest that ACER1 does not play a major role in ceramide degradation in human keratinocytes. In contrast, under undifferentiated conditions, *ASAH1* KO keratinocytes exhibited elevated levels of NS, NdS, NP, AS, and OS ceramides relative to control keratinocytes, with NS and NdS showing the most prominent increases. Upon differentiation, EOS ceramides also showed an increase, in addition to the above ceramide classes. NS, NdS, and OS ceramides showed the most pronounced increases, with levels increased 6.9-fold, 7.8-fold, and 7.0-fold relative to control keratinocytes, respectively. NP and AS ceramides exhibited more moderate increases (4.0-fold and 3.8-fold, respectively), whereas EOS showed a modest 2.2-fold increase. NH ceramide levels were 1.5-fold higher in *ASAH1* KO keratinocytes than in control keratinocytes, but this difference was not statistically significant. No increase was observed in AP or AH ceramides.

Each ceramide class comprises multiple species that differ in the carbon-chain length and degree of unsaturation (saturated or monounsaturated) of the FA (*N*-acyl chain) moiety. The predominant carbon-chain lengths were C16–C26 for NS, NdS, NP, and AS ceramides and C28–C36 for OS and EOS ceramides (Fig. 4*B*) (11). In all these ceramide classes, the levels of all or nearly all species were increased in *ASAH1* KO keratinocytes relative to control cells. However, the degree of increase varied among individual species, with those containing monounsaturated FAs often exhibiting greater increases than those containing saturated FAs. For example, within the NS ceramide class, the species containing C24 FAs showed differential increases: the C24:1 species increased 11.2-fold, whereas the C24:0 species increased 5.6-fold relative to control cells. In the case of C26 FA-containing NS ceramides, the C26:1 species exhibited a 10.2-fold increase, whereas the C26:0 species showed a more modest 3.7-fold elevation. Similar results were obtained for the NdS, NP, AS, and OS classes. However, an exception was observed in the EOS class, where species containing saturated FAs exhibited greater increases than those containing monounsaturated FAs, with C32:0 and C34:0 species showing 2.6-fold and 3.0-fold increases, respectively, and C32:1 and C34:1 species showing 1.8-fold and 2.3-fold increases, all relative to control cells.

In differentiated keratinocytes, ceramides are synthesized in the endoplasmic reticulum and subsequently converted into complex sphingolipids—primarily glucosylceramides and partly sphingomyelins—in the Golgi apparatus. After being stored in lamellar bodies, they are hydrolyzed back to ceramides by β-glucocerebrosidase or acid sphingomyelinase (9). To examine whether ceramide accumulation caused by *ASAH1* deficiency affects the levels of complex sphingolipids, we measured NS and EOS hexosylceramides (glucosylceramides) and NS sphingomyelins in differentiated keratinocytes. All three lipid classes showed comparable levels between control and *ASAH1* KO keratinocytes (Fig. 4*C*).

**Figure 5:**
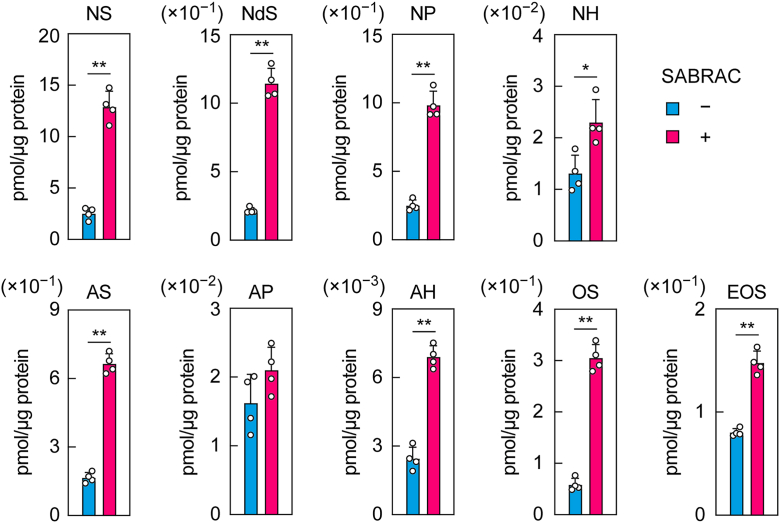
**Ceramide class–dependent effects of the ASAH1 inhibitor SABRAC.** Immortalized human keratinocytes (NHEK/SVTERT3-5) were differentiated and cultured in medium containing 5 μM SABRAC from day 8. On day 14 of differentiation, lipids were extracted from the cells, and the indicated ceramide classes were analyzed *via* LC–MS/MS. Values presented are mean + SD of the quantities of the indicated ceramide classes (n = 4; ∗*p* < 0.05; ∗∗*p* < 0.01; Welch’s *t* test).

To confirm ceramide accumulation resulting from *ASAH1* deficiency, we examined the effect of SABRAC, a specific inhibitor of ASAH1, on ceramide levels. SABRAC was added to the culture medium of differentiated immortalized keratinocytes (NHEK/SVTERT3-5) from day 8 to day 14 of differentiation. Lipids were extracted from the keratinocytes on day 14, and the nine ceramide classes measured above were quantified *via* LC–MS/MS. We found that eight of the classes—excluding AP—were increased in keratinocytes treated with SABRAC relative to those without treatment (Fig. 5). The ceramide classes that showed the greatest increase were NS (5.1-fold), NdS (5.3-fold), and OS (5.2-fold), followed by NP (3.9-fold) and AS (4.0-fold). In contrast, the increases in NH (1.8-fold), AH (2.8-fold), and EOS (1.8-fold) were relatively modest. These results were largely consistent with those observed in *ASAH1* KO keratinocytes (Fig. 4).

### Gene expression changes in *ASAH1* KO keratinocytes

**Figure 6:**
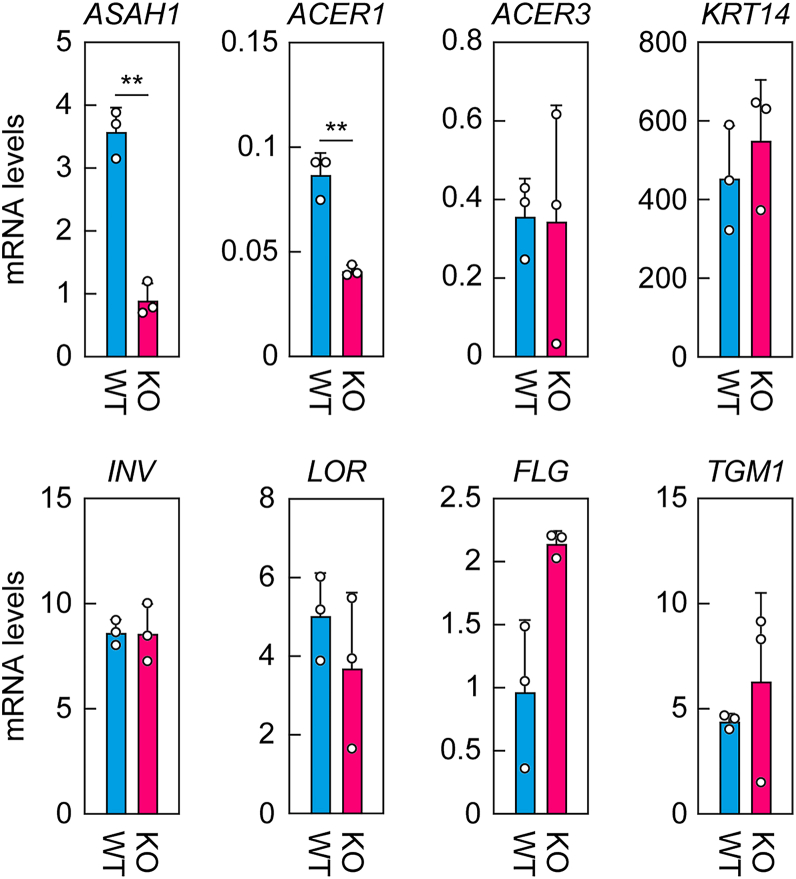
**Gene expression in *ASAH1* KO keratinocytes.** Total RNA was prepared from control and *ASAH1* KO keratinocytes after 14 days of differentiation, and quantitative real-time RT–PCR was performed using gene-specific primers for the indicated genes and the housekeeping gene *HPRT1*. Values presented are mean + SD of gene expression levels relative to *HPRT1* (n = 3; ∗*p* < 0.05; ∗∗*p* < 0.01; Welch’s *t* test).

To investigate gene expression changes in *ASAH1* KO keratinocytes, control and *ASAH1* KO keratinocytes were differentiated for 14 days, after which total RNA was extracted and subjected to quantitative RT–PCR analysis. In *ASAH1* KO keratinocytes, *ASAH1* mRNA levels were reduced to 25% of the control because of gene disruption (Fig. 6). This reduction is likely attributable to nonsense-mediated mRNA decay. The expression levels of *ACER1* were reduced to approximately half of the control in *ASAH1* KO keratinocytes for unknown reasons, whereas *ACER3* expression remained unchanged. The expression levels of *KRT14* (a stratum basale marker) and *INV*, *LOR*, *FLG*, and *TGM1* (stratum granulosum markers) were comparable between control and *ASAH1* KO cells. These results suggest that the differentiation of *ASAH1* KO keratinocytes proceeds normally.

### Substrate specificity of ASAH1 toward different ceramide classes

**Figure 7:**
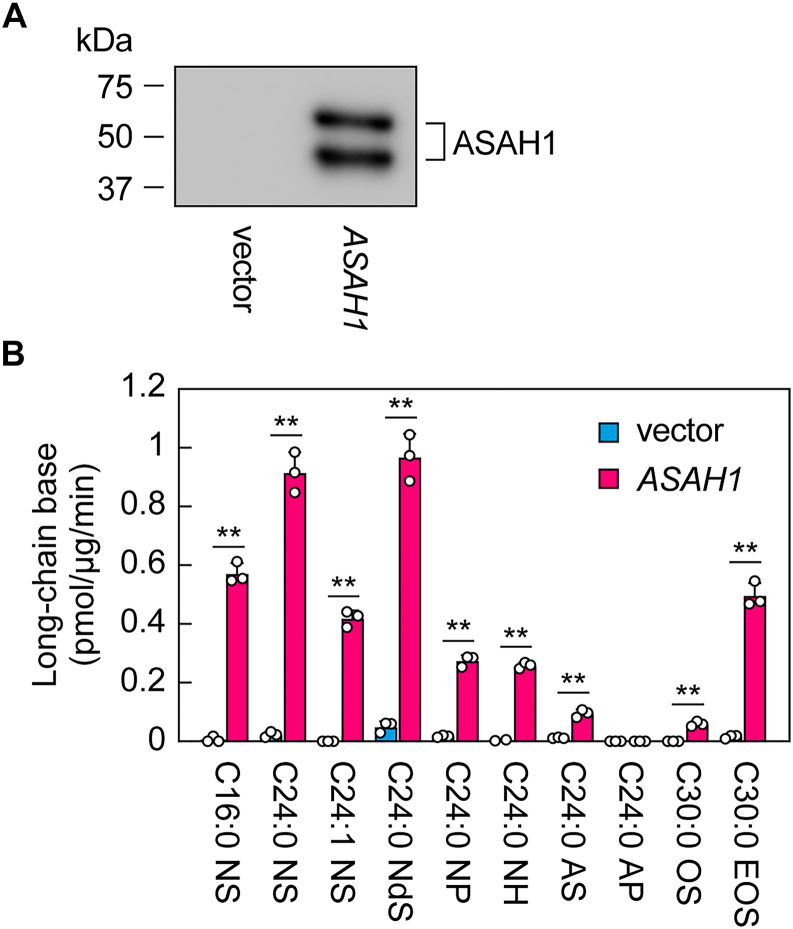
**Ceramide class–specific activity of ASAH1.** HEK 293T cells were transfected with either the pCE-puro 3× FLAG-4 vector or the pCE-puro ASAH1-3× FLAG plasmid. Culture supernatant was collected 48 h after transfection as the enzyme source. *A*, proteins were concentrated *via* precipitation with 5% trichloroacetic acid, followed by SDS-PAGE and immunoblotting using an anti-FLAG antibody. *B*, the indicated ceramide species (final concentration, 5 μM; 600 pmol) were incorporated into liposomes and incubated with the culture supernatant from the pCE-puro ASAH1-3× FLAG plasmid-transfected cells (containing 145 ng ASAH1-3× FLAG) or with supernatant from vector-transfected cells as a negative control. After incubation, lipids were extracted, and the resulting long-chain bases (ceramide degradation products) were quantified *via* LC–MS/MS. Values presented are mean + SD of the quantity of long-chain bases produced per minute by 1 μg of ASAH1-3× FLAG, expressed in picomoles (n = 3; ∗*p* < 0.05; ∗∗*p* < 0.01; Welch’s *t* test). HEK, human embryonic kidney cell line.

*ASAH1* KO or inhibition of ASAH1 by SABRAC led to class-specific changes in quantities (Figs. 4 and 5). A possible explanation for this variation is the different substrate specificity of ASAH1 toward ceramide classes. Therefore, we investigated the enzyme activity of ASAH1 toward various ceramide classes *in vitro*. Although ASAH1 is localized in lysosomes, it is known to be secreted when overexpressed (36). Based on this property, ASAH1 with a C-terminal 3× FLAG tag was overexpressed in human embryonic kidney (HEK) 293T cells, and the culture supernatant was used as the enzyme source in this study. As a control, the culture supernatant from cells transfected with an empty vector was used. Immunoblotting using an anti-FLAG antibody detected two bands at 65 kDa and 48 kDa in the culture supernatant from cells overexpressing ASAH1-3× FLAG (Fig. 7*A*), which may correspond to the precursor and the mature β-subunit of ASAH1, respectively (36, 37, 38).

Next, we performed an *in vitro* ceramidase assay. The culture supernatant obtained was incubated with liposomes containing individual ceramides (C16:0 NS, C24:0 NS, C24:1 NS, C24:0 NdS, C24:0 NP, C24:0 NH, C24:0 AS, C24:0 AP, C30:0 OS, and C30:0 EOS), and the long-chain bases produced by ASAH1 were quantified *via* LC–MS/MS. In a comparison of ceramides containing C24:0 FA, ASAH1 exhibited stronger activity toward NS and NdS, weaker activity toward NP, NH, and AS, and no activity toward AP (Fig. 7*B*). In the case of ceramides containing C30:0 FA, ASAH1 showed stronger activity toward EOS than OS. In a comparison among NS species, ASAH1 showed the strongest activity toward C24:0, followed by C16:0 and C24:1.

**Figure 8:**
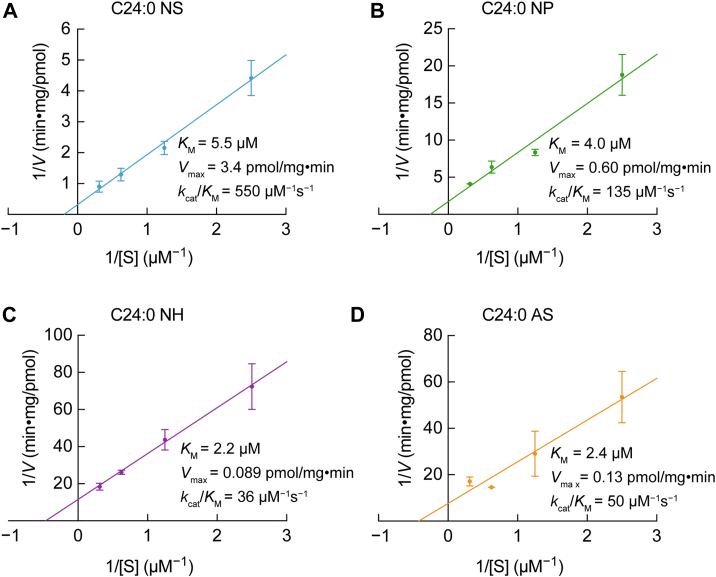
**Enzyme kinetic parameters of ASAH1. HEK 293T cells were transfected with either the pCE-puro 3× FLAG-4 vector or the pCE-puro ASAH1-3× FLAG plasmid.** Culture supernatant was collected 48 h after transfection as the enzyme source. The indicated ceramide species with different classes—C24:0 NS (*A*), C24:0 NP (*B*), C24:0 NH (*C*), and C24:0 AS (*D*)—were incorporated into liposomes at final concentrations of 0.4, 0.8, 1.6, and 3.2 μM and incubated with the culture supernatant at 37 °C for 2 h. After incubation, lipids were extracted, and the resulting long-chain bases (ceramide degradation products) were quantified *via* LC–MS/MS. The data obtained were used to generate Lineweaver–Burk plots by plotting the inverse of reaction velocity (1/*V*) against the inverse of substrate concentration (1/[S]). Values presented are mean ± SD (n = 3). Values of the calculated kinetic parameters (*K*_*M*_, *V*_max_, and *k*_cat_/*K*_*M*_) are also shown. HEK, human embryonic kidney cell line.

To further elucidate the substrate specificity of ASAH1 toward different ceramide classes, we conducted an enzyme kinetic analysis of ASAH1 by measuring its activity under varying concentrations of each substrate. NS, NP, NH, and AS, each containing C24:0 FA, were used as substrates. To determine the kinetic parameters of ASAH1 for each substrate, we plotted the reciprocal of reaction velocity (*V*) against that of substrate concentration ([S]) to construct Lineweaver–Burk plots and obtain the *V*_max_ and *K*_*M*_ values. The specificity constant (*k*_cat_/*K*_M_) was calculated using these values together with the enzyme concentration. ASAH1 exhibited the highest *V*_max_ value toward NS, followed by NP (18% of NS) (Fig. 8). The *V*_max_ values toward AS (4%) and NH (3%) were much lower. The *K*_*M*_ value of ASAH1 was highest toward NS (5.5 μM), but the values for the other substrates—NP (4.0 μM), NH (2.2 μM), and AS (2.4 μM)—were not markedly different. The specificity constant of ASAH1, an indicator of catalytic efficiency, was highest toward NS, followed by NP, AS, and NH in descending order. These results indicate that ASAH1 exhibits substrate specificity toward different ceramide classes, with particularly strong activity toward those containing fewer hydroxyl groups.

## Discussion

Ceramides play a crucial role in the formation of the skin barrier, and reductions in their levels or alterations in ceramide class composition are associated with impaired barrier function and the development of skin disorders, such as atopic dermatitis, xerosis, and ichthyosis (1, 2, 3). In general, levels of biomolecules are regulated by the balance between their synthesis and degradation. However, the extent of ceramide degradation in the epidermis had remained unclear until now. In this study, we found that ASAH1, an acid ceramidase, is involved in the degradation of multiple ceramide classes in keratinocytes, and that *ASAH1* KO or inhibition of ASAH1 by SABRAC led to increases in the levels of these ceramide classes (Figs. 4 and 5).

The major constituents of lipid lamellae are ceramides, cholesterol, and free FAs. Although the reported molar ratios vary among studies, they are generally present in approximately equimolar amounts, or one component may be up to about twice as abundant as the others (6, 7, 8). It has been demonstrated that having a higher ceramide content in the stratum corneum is not necessarily desirable for skin health; rather, maintaining an appropriate ratio of ceramides, cholesterol, and free FAs is crucial. This has been shown by studies examining barrier recovery following topical application of these mixtures after skin barrier disruption (39, 40), by experiments investigating the formation of long-periodicity lamellae (41), and by molecular dynamics simulations (42). Therefore, the degradation of ceramides by ASAH1 in differentiated keratinocytes may be important for maintaining an appropriate ratio of ceramides, cholesterol, and free FAs.

The effects of *ASAH1* KO and inhibition of ASAH1 by SABRAC on ceramide accumulation varied among ceramide classes and were more pronounced for ceramides with fewer hydroxyl groups (Figs. 4 and 5). Long-chain bases commonly possess hydroxyl groups at the C-1 and C-3 positions. Phytosphingosine (P) and 6-hydroxysphingosine (H) each carry an additional hydroxyl group at the C-4 and C-6 position, respectively. With respect to FAs, α-hydroxy FA (A) and ω-hydroxy FA (O) have a hydroxyl group at the α- (C-2) and ω-position, respectively. Both NS and NdS, whose levels were markedly increased by *ASAH1* KO and ASAH1 inhibition, possess only the two hydroxyl groups common to all long-chain bases. In contrast, AP and AH, which carry additional hydroxyl groups on both the long-chain base and FA moieties and thus have four hydroxyl groups in total, were minimally affected or unaffected by *ASAH1* KO or ASAH1 inhibition. Most of the ceramides with three hydroxyl groups were affected to an intermediate extent. One major reason for these class-specific differences in the effects of *ASAH1* KO and ASAH1 inhibition was revealed by *in vitro* enzymatic analyses to be the substrate specificity of ASAH1 (Figs. 7 and 8), showing that ASAH1 exhibited strong activity toward ceramide classes with fewer hydroxyl groups but low activity toward those with more.

The structure of ASAH1 has been determined through X-ray crystallography, and a binding model for ceramide has been proposed based on this structure (43). According to the model, the substrate-binding pocket of ASAH1 consists of a hydrophilic region formed by polar amino acid residues (Cys-143, Asp-162, Asn-320, Arg-333; with Cys-143 functioning as the catalytic residue) and a hydrophobic cavity formed by hydrophobic amino acid residues, where the hydrophilic region interacts with the polar groups common to ceramides. The additional hydroxyl groups present in ceramide classes that are weak substrates for ASAH1 increase the polarity of the ceramide hydrophobic chain, thereby possibly weakening hydrophobic interactions with the hydrophobic cavity. Alternatively, these additional hydroxyl groups may reduce the efficiency of the reaction catalyzed by ASAH1 by competing with the polar groups common to ceramides for interaction with polar amino acid residues in the substrate-binding pocket.

Most ceramides present in typical tissues are NS, which possess only two hydroxyl groups. In contrast, the human stratum corneum contains substantial quantities of ceramides with three or four hydroxyl groups, including NP and NH (three hydroxyls) and AP and AH (four hydroxyls), presenting the following percentages of total free ceramides: NP, 29%; NH, 23%; AP, 6%; and AH, 9% (11). Since strong lipid–lipid interactions are required for the lipid lamellae to form a robust barrier, hydrogen bonding mediated by these hydroxyl groups is likely to be important. To maintain a high proportion of ceramide classes with three or four hydroxyl groups in the stratum corneum, humans may have adopted a strategy of reducing their degradation rather than increasing their synthesis.

ASAH1 can function both intracellularly (in lysosomes) within keratinocytes from the stratum basale through the stratum granulosum and extracellularly (in lipid lamellae) within the stratum corneum (44, 45). In the present study, however, we interpret our observations as reflecting the intracellular function of ASAH1. This interpretation is supported by the finding that ceramides accumulate in *ASAH1* KO keratinocytes even under undifferentiated conditions, which represent the stratum basale, and not only under differentiation conditions (Fig. 4*A*). Furthermore, our two-dimensional culture system maintains a neutral extracellular pH, a condition under which ASAH1 exhibits low enzymatic activity. However, *in vivo*, ASAH1 may also contribute to ceramide degradation extracellularly within the stratum corneum.

While the differential accumulation of ceramide classes resulting from *ASAH1* KO or ASAH1 inhibition can largely be attributed to the substrate specificity of ASAH1, this does not account for all the observed effects. For example, although ASAH1 exhibits relatively strong activity toward the acylceramide EOS (Fig. 7), the impact of its KO/inhibition on EOS accumulation was limited (Figs. 4 and 5). This suggests that factors other than ASAH1 enzymatic activity may contribute to the stability of EOS. One could be the efficiency of ceramide transport to lysosomes, where ASAH1 is localized. Consequently, EOS may be less efficiently transported to lysosomes than other ceramide classes. Further, even within the same ceramide class, the extent of accumulation in *ASAH1* KO keratinocytes varied among species with different FA moieties. For example, within the NS class, monounsaturated species such as C24:1 showed greater accumulation than saturated species such as C24:0 (Fig. 4). In contrast, *in vitro* assays revealed that ASAH1 exhibited greater activity toward C24:0 NS than C24:1 NS (Fig. 7). This discrepancy also suggests the involvement of factors other than enzymatic activity, such as differences in transport efficiency. In differentiated keratinocytes or within the stratum corneum *in vivo*, ceramide degradation by ASAH1 is facilitated by saposin D (46). However, saposin D was not included in our *in vitro* assay. It is possible that the presence of saposin D in differentiated keratinocytes as well as *in vivo* partially modulates ASAH1 substrate specificity.

Long-chain bases generated by ASAH1 in lysosomes are either reutilized for ceramide synthesis in the endoplasmic reticulum or converted into acyl-CoAs through intermediates such as long-chain base 1-phosphates, fatty aldehydes, and FAs, which subsequently serve as precursors for sphingolipid and other lipid synthesis or as substrates for β-oxidation (47). In the latter case, representing the ceramide degradation pathway, dihydrosphingosine (dS) and sphingosine (S) are converted into acyl-CoAs with two fewer carbons than their original long-chain bases, whereas phytosphingosine (P) undergoes additional α-oxidation to yield acyl-CoAs with three fewer carbons (48, 49, 50). In contrast, long-chain bases generated by ASAH1 in the extracellular lipid lamellae may act as antimicrobial agents (51, 52).

This study revealed that many ceramide classes are actively degraded in the epidermis, which may provide a basis for therapeutic development. For instance, in atopic dermatitis, ceramide levels are known to be reduced (53), and an ASAH1 inhibitor may promote the recovery of skin barrier function by increasing ceramide levels. In addition to atopic dermatitis, Sjögren–Larsson syndrome (SLS), a neurocutaneous disorder associated with abnormalities in ceramide degradation (48, 54), may be considered a potential clinical target for ASAH1 inhibition. In the ceramide degradation pathway, the enzyme responsible for converting fatty aldehydes into FAs is fatty aldehyde dehydrogenase ALDH3A2 (48), whose gene is the causative gene of SLS (55). In SLS, accumulated reactive fatty aldehydes are believed to react with proteins that are essential for neural and cutaneous functions, thereby impairing their activity and contributing to the pathogenesis of the condition (54, 55, 56). Inhibition of ASAH1, which blocks the upstream step of the ceramide degradation pathway, may suppress the production of downstream fatty aldehydes and help to alleviate the symptoms of SLS. Future studies into ceramidase inhibitors may lead to effective treatments and eventual clinical application for atopic dermatitis and SLS.

## Experimental procedures

### Cells

Human primary keratinocytes (HPEKp; CELLnTEC Advanced Cell Systems) were cultured using CnT-Prime Epithelial Culture Medium (CELLnTEC Advanced Cell Systems). Immortalized human keratinocytes (NHEK/SVTERT3-5; Evercyte) were cultured in either CnT-Prime Epithelial Culture Medium or EpiLife (Thermo Fisher Scientific) supplemented with 100 μg/ml G418 (InvivoGen). Cells were cultured on collagen-coated dishes (Iwaki) under conditions of 5% CO_2_ at 37 °C. To induce differentiation, cells at 100% confluence were subjected to a medium change to CnT-Prime 3D Barrier Culture Medium (CELLnTEC Advanced Cell Systems) and subsequently cultured for 7 (HPEKp cells) or 14 days (NHEK/SVTERT3-5 cells) with medium changes performed every 3 days. Transfection using NHEK/SVTERT3-5 cells was performed in Keratinocyte Growth Medium-2 (Lonza) using ViaFect Transfection Reagent (Promega), according to the manufacturer’s instructions. HEK 293T cells were cultured in Dulbecco’s modified Eagle's medium (D6429; Merck) supplemented with 10% fetal bovine serum (Thermo Fisher Scientific), 100 units/ml penicillin, and 100 μg/ml streptomycin (Merck). Transfection was performed using Lipofectamine Transfection Reagent with PLUS Reagent (Thermo Fisher Scientific), following the manufacturer’s protocol.

### Generation of ceramidase KO keratinocytes

*ACER1* KO and *ASAH1* KO keratinocytes were generated using NHEK/SVTERT3-5 cells *via* the CRISPR–Cas9 system with the pYU417 vector expressing the Cas9 D10A mutant nuclease (Cas9 nickase) (57), as described previously (54). Guide RNAs targeted two distinct 20-bp sequences adjacent to protospacer-adjacent motif sites located in exon 2 of *ACER1* and exon 3 of *ASAH1*, respectively. The oligonucleotide pairs used for constructing the targeting vectors were as follows: ACER1 KO-F1/-R1 and ACER1 KO-F2/-R2 for *ACER1* KO and ASAH1 KO-F1/-R1 and ASAH1 KO-F2/-R2 for *ASAH1* KO (Table S3). Genotyping was performed using genomic DNA and the following primers: ACER1 GT-F1/-R1/-R2 for *ACER1* KO and ASAH1 GT-F1/-R1/-R2 for *ASAH1* KO (Table S3).

### RNA sequencing

Human primary keratinocytes HPEKp (both undifferentiated and differentiated for 7 days) grown in 12-well plates were washed twice with 0.5 ml of PBS. Total RNA was then prepared using the NucleoSpin RNA II Kit (Takara Bio) according to the manufacturer’s instructions. The quality of the RNA obtained was assessed using an Agilent 2100 Bioanalyzer (Agilent Technologies). DNA libraries were prepared using the Illumina TruSeq RNA Sample Prep Kit v2 (Illumina) according to the manufacturer’s protocol. Sequencing was performed using the NovaSeq 6000 platform (Illumina), generating 150 bp paired-end reads with a total yield of 6 Gb per sample. Quality control of the sequencing reads was performed using FastQC (version 0.11.8) (58). Adapter sequences and low-quality bases were trimmed using Trimmomatic (version 0.39) (59). The trimmed reads were then aligned to the reference genome using STAR (version 2.7.0c) (60). The number of reads mapped to known exon regions was quantified using featureCounts (version 1.6.4) (61) and transcript abundance was calculated as transcripts per million. Differential expression analysis was conducted using DESeq2 (version 1.22.2) (62) to obtain normalized mean expression values (baseMean) and fold changes in expression levels.

### Quantitative real-time RT–PCR

Immortalized human keratinocytes NHEK/SVTERT3-5 cultured in 12-well plates were washed twice with 0.5 ml of PBS. Total RNA was then prepared using the NucleoSpin RNA II Kit (Takara Bio), followed by conversion to complementary DNA (cDNA) using the PrimeScript II first Strand cDNA Synthesis Kit (Takara Bio), both according to the manufacturer’s protocols. Real-time RT–PCR was performed using the cDNA obtained as a template, gene-specific primer sets (RT-F and RT-R primers; Table S3), and the KOD SYBR quantitative PCR Mix (Toyobo) on the CFX96 Touch Real-Time PCR Detection System (Bio-Rad Laboratories) under the following conditions: initial denaturation at 98 °C for 2 min, followed by 40 cycles of 98 °C for 10 s, 60 °C for 10 s, and 68 °C for 30 s. Gene expression levels were normalized to *HPRT1* (hypoxanthine phosphoribosyltransferase 1).

### Lipid extraction from keratinocytes

Keratinocytes cultured in 12-well plates were washed twice with 0.5 ml of PBS, suspended in 0.2 ml of PBS, collected using a cell scraper, and transferred into glass tubes using a cell scraper. After centrifugation of the cells (4000*g*, room temperature, 3 min), the cell pellet was treated with 375 μl of chloroform/methanol (1:2, v/v) containing internal standards: nine deuterium(*d*_9_)-labeled ceramides—*N*-palmitoyl(*d*_9_)-D-*erythro*-sphingosine (*d*_9_-C16:0 NS; 20 pmol), *N*-palmitoyl(*d*_9_)-D-*erythro*-dihydrosphingosine (*d*_9_-C16:0 NdS; 10 pmol), *N*-palmitoyl(*d*_9_)-D-*ribo*-phytosphingosine (*d*_9_-C16:0 NP; 5 pmol), *N*-palmitoyl(*d*_9_)-6-(*R*)-hydroxysphingosine (*d*_9_-C16:0 NH; 2 pmol), *N*-(2'-(*R*)-hydroxypalmitoyl(*d*_9_))-D-*erythro*-sphingosine (*d*_9_-C16:0 AS; 10 pmol), *N*-(2'-(*R*)-hydroxypalmitoyl(*d*_9_))-D-*erythro*-dihydrosphingosine (*d*_9_-C16:0 AdS; 5 pmol), *N*-(2'-(*R*)-hydroxypalmitoyl(*d*_9_))-D-*ribo*-phytosphingosine (*d*_9_-C16:0 AP; 2.5 pmol), *N*-(2'-(*R*)-hydroxypalmitoyl(*d*_9_))-6-(*R*)-hydroxysphingosine (*d*_9_-C16:0 AH; 1 pmol), and *N*-(26-oleoyloxy(*d*_9_) hexacosanoyl)-D-*erythro*-sphingosine (C26:0/*d*_9_-C18:1 EOS; 10 pmol) (Avanti Research). After sonication in a sonicator bath (room temperature, 5 min), the samples were centrifuged (4000*g*, room temperature, 3 min), and the supernatant was collected. The remaining pellet was used for protein quantification with the BCA Protein Assay Kit (Thermo Fisher Scientific). The supernatant was then mixed with 125 μl of chloroform and 225 μl of water, followed by centrifugation (4000*g*, room temperature, 3 min) for phase separation. The lower layer (organic phase) was collected, dried, and stored at −30 °C until analysis. For LC–MS/MS analysis, the dried lipids were suspended in chloroform/methanol (1:2, v/v) and diluted to an appropriate concentration, from which a 5 μl aliquot was subjected to LC–MS/MS (described below).

### Plasmids

The pCE-puro 3× FLAG-4 is a mammalian expression vector designed to produce proteins with a C-terminal 3× FLAG tag (63). The coding sequence (CDS) of human *ASAH1* was amplified *via* PCR from human heart cDNA (Takara Bio) using the primers ASAH1-F and ASAH1-R (Table S3). The amplified CDS was cloned into the TA cloning vector pGEM-T Easy (Promega). After confirming the nucleotide sequence *via* Sanger sequencing, the *ASAH1* CDS was excised with the restriction enzymes MfeI and BamHI and inserted into the EcoRI–BamHI site of pCE-puro 3× FLAG-4 to generate pCE-puro ASAH1-3× FLAG.

### *In vitro* ceramidase assay

HEK 293T cells cultured in 6-well plates were transfected with either the pCE-puro 3× FLAG-4 vector or the pCE-puro ASAH1-3× FLAG plasmid in serum-free OPTI-MEM I medium (Thermo Fisher Scientific). Three hours after transfection, the medium was replaced with fresh OPTI-MEM I, and the medium was collected 45 h later. The medium collected was centrifuged (20,000*g*, room temperature, 1 min), and the supernatant was used as the enzyme source. A portion of the culture supernatant was concentrated *via* precipitation with 5% trichloroacetic acid and subjected to immunoblotting (described below) to confirm protein expression. The quantity of ASAH1-3× FLAG protein was determined using a standard protein, 3× FLAG-maltose-binding protein, which was overexpressed in *Escherichia coli* and purified with amylose resin (New England Biolabs).

Each ceramide substrate was incorporated into liposomes as follows. A mixture of 60 μl of 5 mg/ml 1-palmitoyl-2-oleoyl-*sn*-glycero-3-phosphocholine (16:0/18:1 phosphatidylcholine; Avanti Research), dissolved in chloroform/methanol (1:1, v/v), and 40 μl of each ceramide species at 100 μM—*N*-palmitoyl-d-*erythro*-sphingosine (C16:0 NS; Avanti Research), *N*-lignoceroyl-d-*erythro*-sphingosine (C24:0 NS; Avanti Research), *N*-nervonoyl-d-*erythro*-sphingosine (C24:1 NS; Avanti Research), *N*-lignoceroyl-d-*erythro*-dihydrosphingosine (C24:0 NdS; Avanti Research), *N*-lignoceroyl-d-*ribo*-phytosphingosine (C24:0 NP; Avanti Research), *N*-lignoceroyl-6-(*R*)-hydroxysphingosine (C24:0 NH; Avanti Research), *N*-(2'-(*R*)-hydroxylignoceroyl)-d-*erythro*-sphingosine (C24:0 AS; Avanti Research), *N*-(2'-(*R*)-hydroxylignoceroyl)-d-*ribo*-phytosphingosine (C24:0 AP; Avanti Research), *N*-(30-linoleoyloxy-triacontanoyl)-d-*erythro*-sphingosine (C30:0 EOS; Cayman Chemical), and *N*-ω-hydroxytriacontanoyl-d-*erythro*-sphingosine (C30:0 OS; Cayman Chemical)—was combined and dried. Samples were suspended in 200 μl of 4× assay buffer (0.8 M NaOH/citric acid [pH 4.3], 1.2 M NaCl, and 8 mM DTT), incubated at 50 °C for 1 h to allow hydration, and subsequently sonicated in a sonicator bath (10 s, twice). A 30 μl aliquot of each liposome was mixed with 10 μl of ASAH1-containing culture supernatant (equivalent to 145 ng of ASAH1) and 80 μl of water, and the mixture was incubated at 37 °C for 2 h. After the reaction, 120 μl of methanol and 120 μl of chloroform were added, and the mixture was mixed. The reaction mixture was centrifuged (9000*g*, room temperature, 3 min), and the lower layer was collected, dried, and stored at −30 °C until analysis. For LC–MS/MS analysis, the dried sample was suspended in 100 μl of chloroform/methanol (1:2, v/v), and 5 μl was subjected to LC–MS/MS analysis (described below).

### LC–MS/MS

LC–MS/MS analysis was performed using a Xevo TQ-XS triple quadrupole (Q) mass spectrometer coupled with ultraperformance LC (Waters). Ceramide analysis was conducted using an ACQUITY UPLC CSH C18 reversed-phase column (1.7 μm particle size, 2.1 mm inner diameter, 100 mm length; Waters) under the following conditions: column temperature of 55 °C, flow rate of 0.3 ml/min, and a binary solvent system consisting of solvent A (acetonitrile/water [3:2, v/v] with 5 mM ammonium formate) and solvent B (2-propanol/acetonitrile [9:1, v/v] with 5 mM ammonium formate). The gradient program was as follows: 0 min, 40% B; 0 to 18 min, linear gradient to 100% B; 18 to 23 min, 100% B; 23 to 23.1 min, step to 40% B; and 23.1 to 25 min, 40% B. Long-chain base analysis was performed using the same column under the following conditions: column temperature of 40 °C, flow rate of 0.3 ml/min, and a binary solvent system consisting of solvent C (water containing 0.2% formic acid) and solvent D (acetonitrile containing 0.2% formic acid). The gradient program was as follows: 0 to 1 min, 10% D; 1 to 4 min, linear gradient to 40% D; 4 to 10 min, step to 75% D; 10 to 12 min, step to 100% D; and 12 to 27 min, 100% D. Lipids were ionized *via* electrospray ionization, and positive ions were detected in multiple reaction monitoring mode. The *m/z* values of precursor ions in Q1 and product ions in Q3, as well as the collision energy settings used in multiple reaction monitoring mode, are listed in Tables S4–S7. Data analysis was performed using MassLynx software (Waters). Lipid quantities were calculated based on the ratio of the peak area of each ion to that of the corresponding internal (for ceramides) or external standards (for long-chain bases, hexosylceramides, and sphingomyelins). For long-chain bases, different external standards were used for each class, all purchased from Avanti Research. The external standard for hexosylceramides was glucosylceramide-*d*_3_ (d18:1/*d*_3_-C16:0) and that for sphingomyelins was sphingomyelin-*d*_9_ (d18:1/*d*_9_-C16:0), both obtained from Cayman. Here, d18:1 represents the number of hydroxyl groups (d, dihydroxy), the carbon chain length, and the number of double bonds of the long-chain moiety. Since *d*_9_-labeled OS ceramides are not commercially available, their quantification was performed using a *d*_9_-labeled AS ceramide standard.

### ASAH1 inhibitor treatment of keratinocytes

On day 8 of differentiation, immortalized human keratinocytes (NHEK/SVTERT3-5) cultured in 12-well plates were treated with either 5 μM SABRAC (Cayman Chemical) or 0.1% dimethyl sulfoxide (v/v) as a control. The culture medium was replaced every 2 days. On day 14 of differentiation, lipids were extracted from the keratinocytes, and ceramides were quantified *via* LC–MS/MS as described above.

### Immunoblotting

Immunoblotting was performed essentially as previously described (64). Anti-FLAG monoclonal antibody (M2; 1 μg/ml; Merck) was used as the primary antibody, and horseradish peroxidase–linked anti-rabbit IgG F(ab')_2_ fragment (1:7500 dilution; Cytiva) was used as the secondary antibody. Chemiluminescent detection was carried out as previously described (65).

### Statistics

Data are presented as mean + SD or mean ± SD. Welch’s unpaired two-tailed *t* test was performed using Microsoft Excel (Microsoft) or Prism (Dotmatics), and Dunnett’s test was performed using SAS (SAS Institute). A *p* value of less than 0.05 was considered statistically significant.

## Data availability

All data generated or analyzed during this study are contained within the article.

## Supporting information

This article contains supporting information (Tables S1–S7).

## Conflict of interest

The authors declare that they have no conflicts of interest with the contents of this article.
