# Supplementary material for: Acid ceramidase ASAH1 is a key regulator of epidermal ceramide levels and composition
Source: J Biol Chem. 2026 Jan 20;302(3):111178. doi: 10.1016/j.jbc.2026.111178 (PMC12907855; doi:10.1016/j.jbc.2026.111178)
Supplement: Supporting information (Tables S2-S7) [file mmc2.pdf]

Supporting information

Acid ceramidase ASAH1 is a key regulator of epidermal ceramide levels and composition

Wakana Nobumoto, Tatsuro Naganuma, Nana Nozaka, Yusuke Ohno, Koki Nojiri, and Akio Kihara

This file includes:

Table S2. Gene information and TPM ratios from RNA sequencing

Table S3. Oligonucleotides and primers used in this study

Table S4. MS/MS settings for ceramides

Table S5. MS/MS settings for long-chain bases

Table S6. MS/MS settings for hexosylceramides

Table S7. MS/MS settings for sphingomyelins

Table S2. Gene information and TPM ratios from RNA sequencing

Category	Gene symbol	Full gene name	TPM Ratio (Day 7/Day 0)
Acylceramides	*ELOVL1*	ELOVL fatty acid elongase 1	3.2
	ELOVL4	ELOVL fatty acid elongase 4	51.0
	CYP4F22	Cytochrome P450 family 4 subfamily F member 22	1183.0
	SLC27A4	Solute carrier family 27 member 4	6.4
	CERS3	Ceramide synthase 3	10.2
	PNPLA1	Patatin like phospholipase domain containing 1	1201.0
	ABHD5	Abhydrolase domain containing 5	4.6
Protein-bound ceramides	*UGCG*	UDP-glucose ceramide glucosyltransferase	2.4
	ABCA12	ATP binding cassette subfamily A member 12	30.3
	ALOX12B	Arachidonate 12-lipoxygenase, 12R type	409.6
	ALOXE3	Arachidonate epidermal lipoxygenase 3	24.4
	SDR9C7	Short chain dehydrogenase/reductase family 9C member 7	686.0
	EPHX2	Epoxide hydrolase 2	0.6
	EPHX3	Epoxide hydrolase 3	334.2
	GBA1	Glucosylceramidase beta 1	14.2
Sphingolipid metabolism	*SPTLC1*	Serine palmitoyltransferase long chain base subunit 1	0.8
	SPTLC2	Serine palmitoyltransferase long chain base subunit 2	1.2
	SPTLC3	Serine palmitoyltransferase long chain base subunit 3	18.3
	SPTSSA	Serine palmitoyltransferase small subunit A	0.5

SPTSSB	Serine palmitoyltransferase small subunit B	108.8
ORMDL1	ORMDL sphingolipid biosynthesis regulator 1	0.3
ORMDL2	ORMDL sphingolipid biosynthesis regulator 2	1.1
ORMDL3	ORMDL sphingolipid biosynthesis regulator 3	0.8
KDSR	3-Ketodihydrosphingosine reductase	0.6
CERS2	Ceramide synthase 2	0.2
CERS4	Ceramide synthase 4	3.7
CERS5	Ceramide synthase 5	0.2
CERS6	Ceramide synthase 6	0.4
CERT1	Ceramide transporter 1	1.4
DEGS1	Delta 4-desaturase, sphingolipid 1	1.6
DEGS2	Delta 4-desaturase, sphingolipid 2	475.3
FADS3	Fatty acid desaturase 3	0.2
FA2H	Fatty acid 2-hydroxylase	22.9
SGMS1	Sphingomyelin synthase 1	0.8
SGMS2	Sphingomyelin synthase 2	0.9
GBA2	Glucosylceramidase beta 2	1.5
SMPD1	Sphingomyelin phosphodiesterase 1	3.5
SMPD2	Sphingomyelin phosphodiesterase 2	1.5
SMPD3	Sphingomyelin phosphodiesterase 3	524.9
SMPD4	Sphingomyelin phosphodiesterase 4	0.3
ALDH3A2	Aldehyde dehydrogenase 3 family member A2	1.3
ALDH3B2	Aldehyde dehydrogenase 3 family member B2	298.7
ASAH1	*N*-acylsphingosine amidohydrolase 1	0.7
ASAH2	*N*-acylsphingosine amidohydrolase 2	0.3
ACER1	Alkaline ceramidase 1	1022.4

	ACER2	Alkaline ceramidase 2	0.9
	ACER3	Alkaline ceramidase 3	0.2
	SPHK1	Sphingosine kinase 1	0.2
	SPHK2	Sphingosine kinase 2	0.7
	SGPL1	Sphingosine-1-phosphate lyase 1	0.9
	HACL1	2-Hydroxyacyl-CoA lyase 1	0.4
	HACL2	2-Hydroxyacyl-CoA lyase 2	0.6
	SGPP1	Sphingosine-1-phosphate phosphatase 1	0.4
	SGPP2	Sphingosine-1-phosphate phosphatase 2	33.1
	SPNS2	Spinster homolog 2	185.8
Keratinocyte differentiation	*KRT1*	Keratin 1	17043.5
	KRT5	Keratin 5	0.5
	KRT10	Keratin 10	67.5
	KRT14	Keratin 14	0.9
	LOR	Loricrin	157429.4
	FLG	Filaggrin	927.0
	IVL	Involucrin	213.4
	TGM1	Transglutaminase 1	76.0

Table S3. Oligonucleotides and primers used in this study

Name	Sequence
ASAH1-F	5'-TACAATTGGCCACCATGCCGGGCCGGAGTTGCGTCGCC-3'
ASAH1-R	5'-TAGGATCCGCCCAACCTATACAAGGGTCAGGGCAGTCC-3'
ACER1 KO-F1	5'-TGCATCAGGAGCATCATCAGGTTTT-3'
ACER1 KO-R1	5'-CTGATGATGCTCCTGATGCACGGTG-3'
ACER1 KO-F2	5'-CCGCTACATTTACGTTGTCTGTTTT-3'
ACER1 KO-R2	5'-AGACAACGTAAATGTAGCGGCGGTG-3'
ASAH1 KO-F1	5'-GGGTGGTAAGTCAAGATTTAGTTTT-3'
ASAH1 KO-R1	5'-TAAATCTTGACTTACCACCCCGGTG-3'
ASAH1 KO-F2	5'-GCATGAATTGATGCTTGACAGTTTT-3'
ASAH1 KO-R2	5'-TGTCAAGCATCAATTCATGCCGGTG-3'
ACER1 GT-F1	5'-CCGTCTCCCCTATCTGACGAGCTCC-3'
ACER1 GT-R1	5'-TAGACATGAGGTCCTGGAACTCCTG-3'
ACER1 GT-R2	5'-CCTGTTCCCCCCAAGGAAGGAGGGG-3'
ASAH1 GT-F1	5'-TCTACACTGCTGTTAAGACTTTGCC-3'
ASAH1 GT-R1	5'-ATTTAATATAGAATCTCCACGGTCC-3'
ASAH1 GT-F2	5'-CATAGAAAACCAATTCACATGAACC-3'
ASAH1 RT-F	5'-TTGGCCCCAGCCTACTTTATCCTGG-3'
ASAH1 RT-R	5'-TGAGATATTCTCTTGGCTGGTGCGG-3'
ACER1 RT-F	5'-GATGCCTAGCATCTTCGCCTATC-3'
ACER1 RT-R	5'-ATACATGGAGAACAGGCCTATGATC-3'
HPRT1 RT-F	5'-TGCTCGAGATGTGATGAAGGAG-3'
HPRT1 RT-R	5'-ATAGCCCCCCTTGAGCACAC-3'
KRT14 RT-F	5'-CAACAGCGAGCTGGTGCAGAGCGGC-3'
KRT14 RT-R	5'-TAGGTGGCGATCTCCTGCTCCAGCC-3'

INV RT-F	5'-ACACACTGCCAGTGACCCTCTCCCC-3'
INV RT-R	5'-TTCTGCTTTCTGATATTCCTCATGC-3'
LOR RT-F	5'-CGAAGGAGTTGGAGGTGTTT-3'
LOR RT-R	5'-ACTGGGGTTGGGAGGTAGTT-3'
FLG RT-F	5'-TCCTCTCACCGCGATACAGC-3'
FLG RT-R	5'-ACCTTTTTGCCTTTCAGTGCC-3'
TGM1 RT-F	5'-GACCCCCGCAATGAGATCTACATCC-3'
TGM1 RT-R	5'-TCCATTGTCATCCAGGGAGTTCACC-3'
CYP4F22 RT-F	5'-AGCGGTCTCCCTTGATATGTT-3'
CYP4F22 RT-R	5'-TAGTGGTGCAAGCGATACTGG-3'
FATP4 RT-F	5'-GCCCTGGACCCAGGTGGGATTCTCC-3'
FATP4 RT-R	5'-GAGTACTCATCCAGCTGGCGGAAGG-3'
CERS3 RT-F	5'-GTTTAGGAGTCGGCGGAATCAAG-3'
CERS3 RT-R	5'-AAACGCAATTCCAGCAACAGTG-3'

Table S4. MS/MS settings for ceramides

Ceramide class	FA chain	Precursor ion(s) (Q1)		Product ion (Q3)	Cone voltage (V)	Collision energy (eV)
		$[M-H_2O+H]^+$	$[M+H]^+$			
NS	d_9-C16:0	529.5		264.3	30	20
NS	C16:0	520.5		264.3	30	20
NS	C18:0	548.5		264.3	30	20
NS	C20:0	576.5		264.3	30	20
NS	C22:0	604.6		264.3	30	25
NS	C24:0	632.6		264.3	30	30
NS	C26:0	660.7		264.3	30	30
NS	C28:0	688.7		264.3	30	30
NS	C30:0	716.7		264.3	30	35
NS	C32:0	744.8		264.3	30	40
NS	C34:0	772.8		264.3	30	40
NS	C36:0	800.8		264.3	30	40
NS	C16:1	518.5		264.3	30	20
NS	C18:1	546.5		264.3	30	20
NS	C20:1	574.5		264.3	30	20
NS	C22:1	602.6		264.3	30	25
NS	C24:1	630.6		264.3	30	30
NS	C26:1	658.7		264.3	30	30
NS	C28:1	686.7		264.3	30	30
NS	C30:1	714.7		264.3	30	35
NS	C32:1	742.8		264.3	30	35
NS	C34:1	770.8		264.3	30	40
NS	C36:1	798.8		264.3	30	40
NDS	d_9-C16:0		549.5	284.3	30	25
NDS	C16:0		540.5	284.3	30	20
NDS	C18:0		568.6	284.3	30	20
NDS	C20:0		596.6	284.3	30	20

NDS	C22:0	624.6	284.3	30	25
NDS	C24:0	652.6	284.3	30	32
NDS	C26:0	680.7	284.3	30	30
NDS	C28:0	708.7	284.3	30	30
NDS	C30:0	736.7	284.3	30	35
NDS	C32:0	764.8	284.3	30	40
NDS	C34:0	792.8	284.3	30	40
NDS	C36:0	820.8	284.3	30	40
NDS	C16:1	538.5	284.3	30	20
NDS	C18:1	566.6	284.3	30	20
NDS	C20:1	594.6	284.3	30	20
NDS	C22:1	622.6	284.3	30	25
NDS	C24:1	650.6	284.3	30	30
NDS	C26:1	678.7	284.3	30	30
NDS	C28:1	706.7	284.3	30	30
NDS	C30:1	734.7	284.3	30	35
NDS	C32:1	762.8	284.3	30	35
NDS	C34:1	790.8	284.3	30	40
NDS	C36:1	818.8	284.3	30	40
NP	d_9-C16:0	565.5	300.3	30	25
NP	C16:0	556.6	300.3	30	25
NP	C18:0	584.6	300.3	30	25
NP	C20:0	612.6	300.3	30	25
NP	C22:0	640.7	300.3	30	30
NP	C24:0	668.7	300.3	30	30
NP	C26:0	696.7	300.3	30	30
NP	C28:0	724.8	300.3	30	35
NP	C30:0	752.8	300.3	30	35
NP	C32:0	780.8	300.3	30	35
NP	C34:0	808.9	300.3	30	45
NP	C36:0	836.9	300.3	30	45
NP	C16:1	554.6	300.3	30	25

NP	C18:1		582.6	300.3	30	25
NP	C20:1		610.6	300.3	30	25
NP	C22:1		638.7	300.3	30	30
NP	C24:1		666.7	300.3	30	30
NP	C26:1		694.7	300.3	30	30
NP	C28:1		722.8	300.3	30	35
NP	C30:1		750.8	300.3	30	35
NP	C32:1		778.8	300.3	30	35
NP	C34:1		806.9	300.3	30	45
NP	C36:1		834.9	300.3	30	45
NH	d_9-C16:0	545.4		280.3	30	20
NH	C16:0	536.5		280.3	30	20
NH	C18:0	564.6		280.3	30	20
NH	C20:0	592.6		280.3	30	20
NH	C22:0	620.6		280.3	30	25
NH	C24:0	648.7		280.3	30	25
NH	C26:0	676.7		280.3	30	25
NH	C28:0	704.7		280.3	30	25
NH	C30:0	732.7		280.3	30	30
NH	C32:0	760.8		280.3	30	35
NH	C34:0	788.8		280.3	30	35
NH	C36:0	816.8		280.3	30	35
NH	C16:1	534.5		280.3	30	20
NH	C18:1	562.6		280.3	30	20
NH	C20:1	590.6		280.3	30	20
NH	C22:1	618.6		280.3	30	25
NH	C24:1	646.7		280.3	30	25
NH	C26:1	674.7		280.3	30	25
NH	C28:1	702.7		280.3	30	25
NH	C30:1	730.7		280.3	30	30
NH	C32:1	758.8		280.3	30	30
NH	C34:1	786.8		280.3	30	35

NH	C36:1	814.8		280.3	30	35
AS	d_9-C16:0	545.5		264.3	30	20
AS	C16:0	536.5		264.3	30	20
AS	C18:0	564.6		264.3	30	20
AS	C20:0	592.6		264.3	30	20
AS	C22:0	620.6		264.3	30	25
AS	C24:0	648.6		264.3	30	30
AS	C26:0	676.7		264.3	30	30
AS	C28:0	704.7		264.3	30	30
AS	C30:0	732.7		264.3	30	35
AS	C32:0	760.8		264.3	30	40
AS	C34:0	788.8		264.3	30	40
AS	C36:0	816.8		264.3	30	40
AS	C16:1	534.5		264.3	30	20
AS	C18:1	562.6		264.3	30	20
AS	C20:1	590.6		264.3	30	20
AS	C22:1	618.6		264.3	30	25
AS	C24:1	646.6		264.3	30	30
AS	C26:1	674.7		264.3	30	30
AS	C28:1	702.7		264.3	30	30
AS	C30:1	730.7		264.3	30	35
AS	C32:1	758.8		264.3	30	35
AS	C34:1	786.8		264.3	30	40
AS	C36:1	814.8		264.3	30	40
AP	d_9-C16:0		581.5	300.3	30	25
AP	C16:0		572.6	300.3	30	25
AP	C18:0		600.6	300.3	30	25
AP	C20:0		628.6	300.3	30	25
AP	C22:0		656.7	300.3	30	30
AP	C24:0		684.7	300.3	30	35
AP	C26:0		712.7	300.3	30	35
AP	C28:0		740.8	300.3	30	35

AP	C30:0		768.8	300.3	30	40
AP	C32:0		796.8	300.3	30	45
AP	C34:0		824.9	300.3	30	45
AP	C36:0		852.9	300.3	30	45
AP	C16:1		570.6	300.3	30	25
AP	C18:1		598.6	300.3	30	25
AP	C20:1		626.6	300.3	30	25
AP	C22:1		654.7	300.3	30	30
AP	C24:1		682.7	300.3	30	35
AP	C26:1		710.7	300.3	30	35
AP	C28:1		738.8	300.3	30	35
AP	C30:1		766.8	300.3	30	40
AP	C32:1		794.8	300.3	30	40
AP	C34:1		822.9	300.3	30	45
AP	C36:1		850.9	300.3	30	45
AH	d_9-C16:0	561.5		280.3	30	20
AH	C16:0	552.5		280.3	30	20
AH	C18:0	580.6		280.3	30	20
AH	C20:0	608.6		280.3	30	20
AH	C22:0	636.6		280.3	30	25
AH	C24:0	664.6		280.3	30	30
AH	C26:0	692.7		280.3	30	30
AH	C28:0	720.7		280.3	30	30
AH	C30:0	748.7		280.3	30	35
AH	C32:0	776.8		280.3	30	40
AH	C34:0	804.8		280.3	30	40
AH	C36:0	832.8		280.3	30	40
AH	C16:1	550.5		280.3	30	20
AH	C18:1	578.6		280.3	30	20
AH	C20:1	606.6		280.3	30	20
AH	C22:1	634.6		280.3	30	25
AH	C24:1	662.6		280.3	30	30

AH	C26:1	690.7		280.3	30	30
AH	C28:1	718.7		280.3	30	30
AH	C30:1	746.7		280.3	30	35
AH	C32:1	774.8		280.3	30	35
AH	C34:1	802.8		280.3	30	40
AH	C36:1	830.8		280.3	30	40
OS	C26:0	676.7	694.7	264.3	30	30
OS	C28:0	704.7	722.7	264.3	30	30
OS	C30:0	732.7	750.7	264.3	30	35
OS	C32:0	760.8	778.8	264.3	30	40
OS	C34:0	788.8	806.8	264.3	30	40
OS	C36:0	816.8	834.8	264.3	30	40
OS	C26:1	674.7	692.7	264.3	30	30
OS	C28:1	702.7	720.7	264.3	30	30
OS	C30:1	730.7	748.7	264.3	30	35
OS	C32:1	758.8	776.8	264.3	30	35
OS	C34:1	786.8	804.8	264.3	30	40
OS	C36:1	814.8	832.8	264.3	30	40
EOS	C26:0/d_9-C18:1	949.9	967.9	264.3	46	35
EOS	C26:0/C18:2	938.9	956.9	264.3	46	35
EOS	C28:0/C18:2	967.0	985.0	264.3	46	35
EOS	C30:0/C18:2	995.0	1013.0	264.3	46	40
EOS	C32:0/C18:2	1023.0	1041.0	264.3	46	40
EOS	C34:0/C18:2	1051.1	1069.1	264.3	46	40
EOS	C36:0/C18:2	1079.1	1097.1	264.3	46	45
EOS	C26:1/C18:2	936.9	954.9	264.3	46	35
EOS	C28:1/C18:2	965.0	983.0	264.3	46	35
EOS	C30:1/C18:2	993.0	1011.0	264.3	46	40
EOS	C32:1/C18:2	1021.0	1039.0	264.3	46	40
EOS	C34:1/C18:2	1039.1	1067.1	264.3	46	40
EOS	C36:1/C18:2	1077.1	1095.1	264.3	46	45

Table S5. MS/MS settings for long-chain bases

Long chain base	Precursor ion (Q1)	Product ion(s) (Q3)	Cone voltage (V)	Collision energy (eV)
d_9-d18:1 sphingosine	291.3	271.3	55	15
d18:1 sphingosine	282.3	252.3<br>264.3	55	15
d18:0 dihydrosphingosine	302.4	254.2<br>266.2<br>284.4	55	12
t18:0 phytosphingosine	318.4	282.3<br>300.3	10	14
t18:1 6-hydroxysphingosine	316.4	280.3	10	15

d, dihydroxy; t, trihydroxy.

Table S6. MS/MS settings for hexosylceramides

Hexosylceramide species	Precursor ions (Q1)		Product ion (Q3)	Collision energy (eV)
	$[M-H_2O+H]^+$	$[M+H]^+$		
NS (d18:1/d_3-C16:0)		703.6	264.3	40
NS (d18:1/C16:0)		700.5	264.3	40
NS (d18:1/C18:0)		728.6	264.3	40
NS (d18:1/C20:0)		756.6	264.3	45
NS (d18:1/C22:0)		784.6	264.3	45
NS (d18:1/C24:0)		812.6	264.3	45
NS (d18:1/C26:0)		840.7	264.3	45
NS (d18:1/C28:0)		868.7	264.3	45
NS (d18:1/C16:1)		698.5	264.3	40
NS (d18:1/C18:1)		726.6	264.3	40
NS (d18:1/C20:1)		754.6	264.3	45
NS (d18:1/C22:1)		782.6	264.3	45
NS (d18:1/C24:1)		810.7	264.3	45
NS (d18:1/C26:1)		838.7	264.3	45
NS (d18:1/C28:1)		866.7	264.3	45
EOS (d18:1/C28:0/C18:2)	1129.0	1147.0	264.3	45
EOS (d18:1/C30:0/C18:2)	1157.0	1175.0	264.3	50
EOS (d18:1/C32:0/C18:2)	1185.0	1203.0	264.3	50
EOS (d18:1/C34:0/C18:2)	1213.1	1231.1	264.3	50
EOS (d18:1/C36:0/C18:2)	1241.1	1259.1	264.3	55
EOS (d18:1/C28:1/C18:2)	1127.0	1145.0	264.3	45
EOS (d18:1/C30:1/C18:2)	1155.0	1173.0	264.3	50
EOS (d18:1/C32:1/C18:2)	1183.0	1201.0	264.3	50
EOS (d18:1/C34:1/C18:2)	1211.1	1229.1	264.3	50
EOS (d18:1/C36:1/C18:2)	1239.1	1257.1	264.3	55

d, dihydroxy.

Table S7. MS/MS settings for sphingomyelins

Sphingomyelin species	Precursor ion (Q1) $[M+H]^+$	Product ion (Q3)	Collision energy (eV)
NS (d18:1/*d*$_9$-C16:0)	712.7	184.1	60
NS (d18:1/C16:0)	703.7	184.1	60
NS (d18:1/C18:0)	731.7	184.1	60
NS (d18:1/C20:0)	759.8	184.1	60
NS (d18:1/C22:0)	787.8	184.1	60
NS (d18:1/C24:0)	815.8	184.1	60
NS (d18:1/C26:0)	843.8	184.1	60
NS (d18:1/C28:0)	871.8	184.1	60
NS (d18:1/C16:1)	701.7	184.1	60
NS (d18:1/C18:1)	731.7	184.1	60
NS (d18:1/C20:1)	757.7	184.1	60
NS (d18:1/C22:1)	785.8	184.1	60
NS (d18:1/C24:1)	813.8	184.1	60
NS (d18:1/C26:1)	841.8	184.1	60
NS (d18:1/C28:1)	869.8	184.1	60

d, dihydroxy.
